# Recent Insights on the Maternal Microbiota: Impact on Pregnancy Outcomes

**DOI:** 10.3389/fimmu.2020.528202

**Published:** 2020-10-23

**Authors:** Nicoletta Di Simone, Amparo Santamaria Ortiz, Monia Specchia, Chiara Tersigni, Paola Villa, Antonio Gasbarrini, Giovanni Scambia, Silvia D’Ippolito

**Affiliations:** ^1^Dipartimento di Scienze della Vita e Sanità Pubblica, Università Cattolica del Sacro Cuore, Rome, Italy; ^2^Dipartimento di Scienze della Salute della Donna, del Bambino e di Sanità Pubblica, Fondazione Policlinico Universitario A. Gemelli Istituto di Ricovero e Cura a Carattere Scientifico, Rome, Italy; ^3^Department of Hematology, University Hospital Vinalopo-Torrevieja, Alicante, Spain; ^4^Dipartimento di Scienze Gastroenterologiche, Endocrino-Metaboliche e Nefro-Urologiche, Fondazione Policlinico Universitario A. Gemelli Istituto di Ricovero e Cura a Carattere Scientifico, Rome, Italy

**Keywords:** microbiota, pregnancy, immunity, gut, vagina, endometrium, inflammasome

## Abstract

Hormonal changes during and after pregnancy are linked with modifications in the maternal microbiota. We describe the importance of the maternal microbiota in pregnancy and examine whether changes in maternal microbiotic composition at different body sites (gut, vagina, endometrium) are associated with pregnancy complications. We analyze the likely interactions between microbiota and the immune system. During pregnancy, the gastrointestinal (gut) microbiota undergoes profound changes that lead to an increase in lactic acid–producing bacteria and a reduction in butyrate-producing bacteria. The meaning of such changes needs clarification. Additionally, several studies have indicated a possible involvement of the maternal gut microbiota in autoimmune and lifelong diseases. The human vagina has its own microbiota, and changes in vaginal microbiota are related to several pregnancy-related complications. Recent studies show reduced lactobacilli, increased bacterial diversity, and low vaginal levels of beta-defensin 2 in women with preterm births. In contrast, early and healthy pregnancies are characterized by low diversity and low numbers of bacterial communities dominated by *Lactobacillus*. These observations suggest that early vaginal cultures that show an absence of *Lactobacillus* and polymicrobial vaginal colonization are risk factors for preterm birth. The endometrium is not a sterile site. Resident endometrial microbiota has only been defined recently. However, questions remain regarding the main components of the endometrial microbiota and their impact on the reproductive tract concerning both fertility and pregnancy outcomes. A classification based on endometrial bacterial patterns could help develop a microbiota-based diagnosis as well as personalized therapies for the prevention of obstetric complications and personalized treatments through nutritional, microbiotic, or pharmaceutical interventions.

## Introduction

The human body is host to a community of microorganisms, including viruses, bacteria, and fungi. The bacterial component of this community, the microbiota, is known to influence health given its symbiotic relationship with the human host.

Vertical transmission of bacteria from mother to newborn contributes to developing the microbiota of the infant gastrointestinal (gut); emerging evidence suggests that this influence may begin in utero (1).

Important changes in the maternal gut microbiota have been observed during pregnancy. These changes are associated with an increase in maternal body weight and dietary changes. Physiological maternal metabolic modifications maintain maternal hyperglycemia and provide glucose to the growing fetus (2). The growth of bacterial species that can synthesize glycogen is stimulated in the presence of increased concentrations of glucose (3). In fact, the transcriptomic pattern of the maternal gut microbiota shows microbiotic changes related to hyperglycemia, especially in the third trimester (4).

The maternal gut microbiota may influence the growth of bacteria in the newborn’s gut, affecting its function and the development of the immune system (5). How the microbiota impacts the immune system in the short and long terms is a critical concern. The microbiota is involved in the regulation of T cell expansion, the development and function of macrophages, and neutrophil chemotaxis (6–8). A major contribution to this debate illustrates how the transient colonization of pregnant female mice with engineered *Escherichia coli* may modify the levels of intestinal innate immune composition in mother and offspring. This suggests that gut maternal microorganisms may play a role in the regulation of the immune system of newborns (9). Vitamin synthesis, gut barrier function, and development of the immune system are essential functions of human health that develop alongside the expansion of the gut microbiota (9). Thus, any changes in the maturation of the gut microbiota in the infant may influence future bacterial colonization and the development of the immune system with possible health consequences.

Despite various scholarly contributions on the maternal microbiota in late pregnancy, only empirical evidence can capture changes in the maternal microbiota at this stage. To date, several factors have been known to influence the human microbiota, such as ancestry, antibiotic use, lifestyle, dietary habits, exercise frequency, and body mass index (BMI) (10). This means that there is no unique health or disease indicator related to the microbiota, yet each individual has a different microbiota from that of others. Consequently, evaluating the microbiota in early pregnancy or even before pregnancy may be a useful tool to enable a personalized approach. Demonstration that an altered microbiota may be linked to maternal and fetal complications is an important target in personalized medicine.

## The Maternal Microbiota and Pregnancy Outcomes

### The Gastrointestinal Microbiota

During pregnancy, maternal fat deposition and food intake increase progressively. In the second and third trimesters, maternal metabolic changes include increased gluconeogenesis, lipolysis, and insulin resistance. Such an acquired diabetogenic condition is functional and induces maternal physiological hyperglycemia, which, in turn, increases glucose availability for the growing fetus. Therefore, significant changes in the maternal gut microbiota occur during pregnancy (2, 4, 11). Although scholars have explored the maternal microbiota in the third trimester of pregnancy, data on the changes in maternal microbiota during early pregnancy are scarce (2, 4, 11, 12).

The gut microbiota in the first trimester of pregnancy resembles the microbiota of healthy nonpregnant women (2, 4, 11). Women have unique gut microbiota that can be classified into different classes or enterotypes. In turn, these are characterized by different groups of bacteria (13, 14). Currently, three classes of enterotypes are recognized, each with its dominant group of bacteria: enterotype I, which is characterized by the presence of *Bacteroides*; enterotype II, characterized by *Prevotella*; and enterotype III, dominated by *Ruminococcus*. The three enterotypes have different and specific functions, producing energy from carbohydrates or proteins (13, 14). A different enterotype characterizes each individual and can be modified by various factors, including diet and BMI (15, 16). Recently, Barret et al. (16) analyze the maternal intestinal microbiota in early pregnancy by comparing an omnivorous diet to a vegetarian one. Women consuming a vegetarian diet showed an increase of bacterial clusters involved in lipid synthesis, which suggests alteration of fermentation and presence of bacterial species producing large amounts of short-chain fatty acids (enterotype II). Studies on gut microbiota in a sample of African women detect the prevalence of enterotype II (*Prevotella*). A diet rich in vegetables with low consumption of animal proteins and lipids allows the growth of bacterial clusters, which degrade the mucin-type glycoproteins that cover the gut mucosal layer. Conversely, a European diet rich in animal protein and lipids is associated with enterotype I (*Bacteroides*). This enterotype produces energy from proteins and carbohydrates (13, 14, 16).

An obese state has also been associated with microbiotic composition during gestation (2, 11, 17). Levels of *Bacteroides* and *Staphylococcus* are higher in the feces produced by overweight pregnant women compared to those with a healthy weight (17). Additionally, in overweight and obese pregnant women, insulin and adipokines (adipose tissue–derived cytokines) correlate with alterations in bacterial abundance, confirming an association between the microbiota and the level of metabolic hormones and cytokines in pregnancy (18). The pregestational BMI contributes to an increased risk of obstetric complications through cellular and molecular processes that are poorly understood (19). Normal placental development and pregnancy success are largely dependent on angiogenic and vascular remodeling events that take place within the maternal–fetal interface (20–22). Several populations of leukocytes in the decidual microenvironment control the early stages of trophoblast invasiveness; uterine natural killer cells are the most abundant immune cell subtype within the decidua (23–26). These cells are key players in uterine vascular growth through the production of proangiogenic factors and tissue-remodeling cytokines (25–29). Obesity, accompanied by increased adipose tissue richness in macrophages, T and B lymphocytes, mast cells, and neutrophils, is associated with altered levels of proinflammatory cytokines. In addition, obese women show decreased levels of decidual uterine natural killer cells with reduced production of proangiogenic factors (30, 31).

Several studies support the hypothesis that changes in the gut microbiota during early pregnancy are associated with an increased risk of gestational diabetes and hypertension (32–35). Enriched abundance of *Blautia* and *Ruminococcus* has been observed in patients with diabetes (35). Gomez-Arango et al. (36) find that the abundance of *Odoribacter*, a butyric acid–producing bacterium, is negatively correlated with systolic blood pressure in pregnant women at 16 weeks of pregnancy. Lv et al. (35) find an important association between alterations in gut microbiota (dysbiosis) and early-onset preeclampsia (PE). They show that the composition of gut microbiota in patients with early-onset PE differed significantly from that in healthy pregnant women. They identified that the bacteria associated with PE were also associated with other host morbidities, including obesity, higher incidence of glucose metabolic disorders, proinflammatory states, and intestinal barrier dysfunction. In addition, these microorganisms correlated with host immune parameters, such as interleukin-6 and lipopolysaccharide (LPS), the major component of the outer membrane of Gram-negative bacteria. Overall, these findings suggest that an altered gut microbiota during early pregnancy (by acting on the maternal immune system and affecting the production of proinflammatory cytokines) may be involved in the development of pregnancy-related complications, such as early-onset PE.

**Figure 1:**
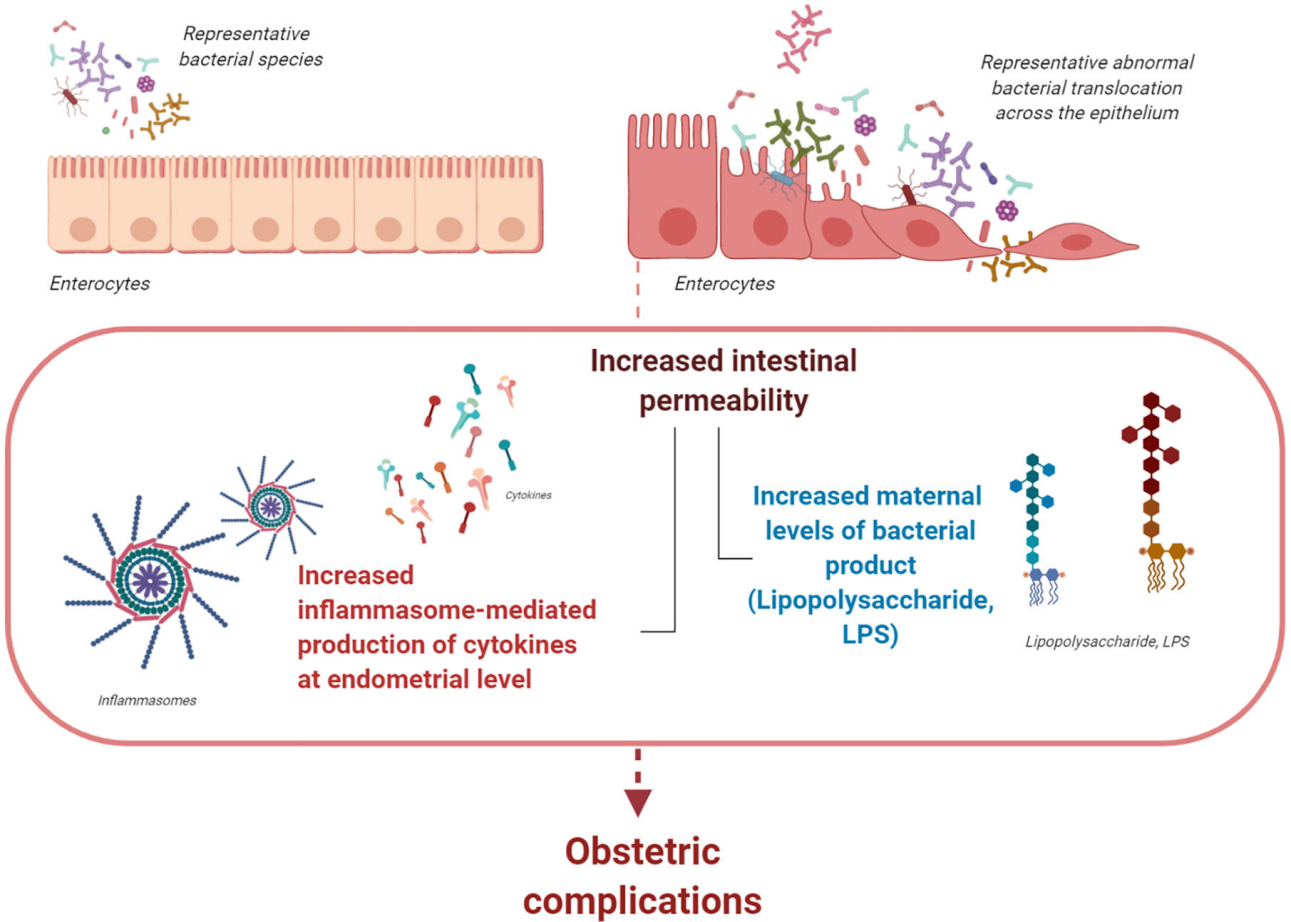
The gastrointestinal (gut) microbiota. During pregnancy, the gut microbiota undergoes profound changes with different enterotypes characterizing each woman. If abnormal bacterial translocation across the epithelium that is associated with increased levels of LPS occurs during early pregnancy, uterine innate immunity and obstetric outcome may be affected. Abnormally increased intestinal permeability during early pregnancy is associated with increased levels of circulating bacterial products and cytokines. Both events might increase inflammasome activation at the endometrial level; consequently, they increase the risk of obstetric complications during early pregnancy (figure created with BioRender.com).

In a recent study, we hypothesized that, if abnormal bacterial translocation across the epithelium occurs early in pregnancy (with LPS being a marker of increased bacterial translocation across the intestinal epithelium), then uterine innate immunity and obstetric outcome may be affected. We find that increased intestinal permeability in early pregnancy is associated with increased maternal levels of LPS, excessive inflammasome-mediated production of cytokines at the endometrial level, and last, increased risk of pregnancy loss (37, 38). Therefore, we suggest that, during early pregnancy, gut bacterial products from the intestinal lumen are translocated into the maternal circulation. This is likely associated with increased intestinal permeability and may increase the risk of obstetric complications (**Figure 1**).

During the third trimester of pregnancy, butyrate-producing bacteria with anti-inflammatory activities decline, whereas bifidobacteria, proteobacteria, and lactic acid–producing bacteria increase (2, 4, 11, 39). Additionally, the maternal gut microbiota grows less actively, reaching a stationary phase accompanied by reduced gut motility and increased intestinal permeability (2, 11, 39). Gastrointestinal modifications in the third trimester concern the host immune system of the gastrointestinal mucosa. Together with changes in metabolic, hormonal, and gastrointestinal permeability, these modifications may increase the diffusion of glucose from the gut epithelium toward the lumen and bacterial translocation. Collectively, these changes impact the composition of the gut microbiota and, consequently, maternal weight gain (4, 39, 40). Some of the proposed mechanisms by which the gut microbiota plays a role in host weight gain during pregnancy include enhanced absorption of glucose and fatty acids, increased fasting-induced adipocyte factor secretion, induction of catabolic pathways, and immune system stimulation (2, 11, 17, 39, 40).

The meaning of modifications in the gut microbiota has been investigated (2, 11, 41, 42). Notably, fecal transplantation of first- and third-trimester fecal microbiotas to germ-free mice revealed that mice transplanted with third-trimester microbiota gained significantly more weight, developed insulin resistance, and had a greater inflammatory response compared to mice transplanted with first trimester-microbiota (11). These findings demonstrate the direct role of microbiotic components in inducing changes in host immunology and metabolism. Interestingly, such modifications resemble those seen in metabolic syndrome, despite occurring in a physiological rather than pathological condition, such as being 7–9 months pregnant.

The gut microbiota during pregnancy is a critical determinant of offspring health (5, 18, 41, 42). Potentially, it determines the development of atopy and autoimmune phenotypes in the offspring (5). The commensal microbiota has a role in regulating host immunity to pathogens and autoimmune responses. Indeed, the microbiota is a source of metabolites and peptide ligands for T cell recognition, known as pathogen-associated molecular patterns (PAMPs), which are recognized by immune receptors. Microbiota-derived metabolites and PAMPs can affect target organs and activate the autoimmune cascade. This does not start after birth but may occur in the womb, engendering a predisposition of the progeny to disease (43). Despite this, the relationship between the immune system, gut microbiota, and metabolism of pregnant women is unclear.

### The Vaginal Microbiota

**Figure 2:**
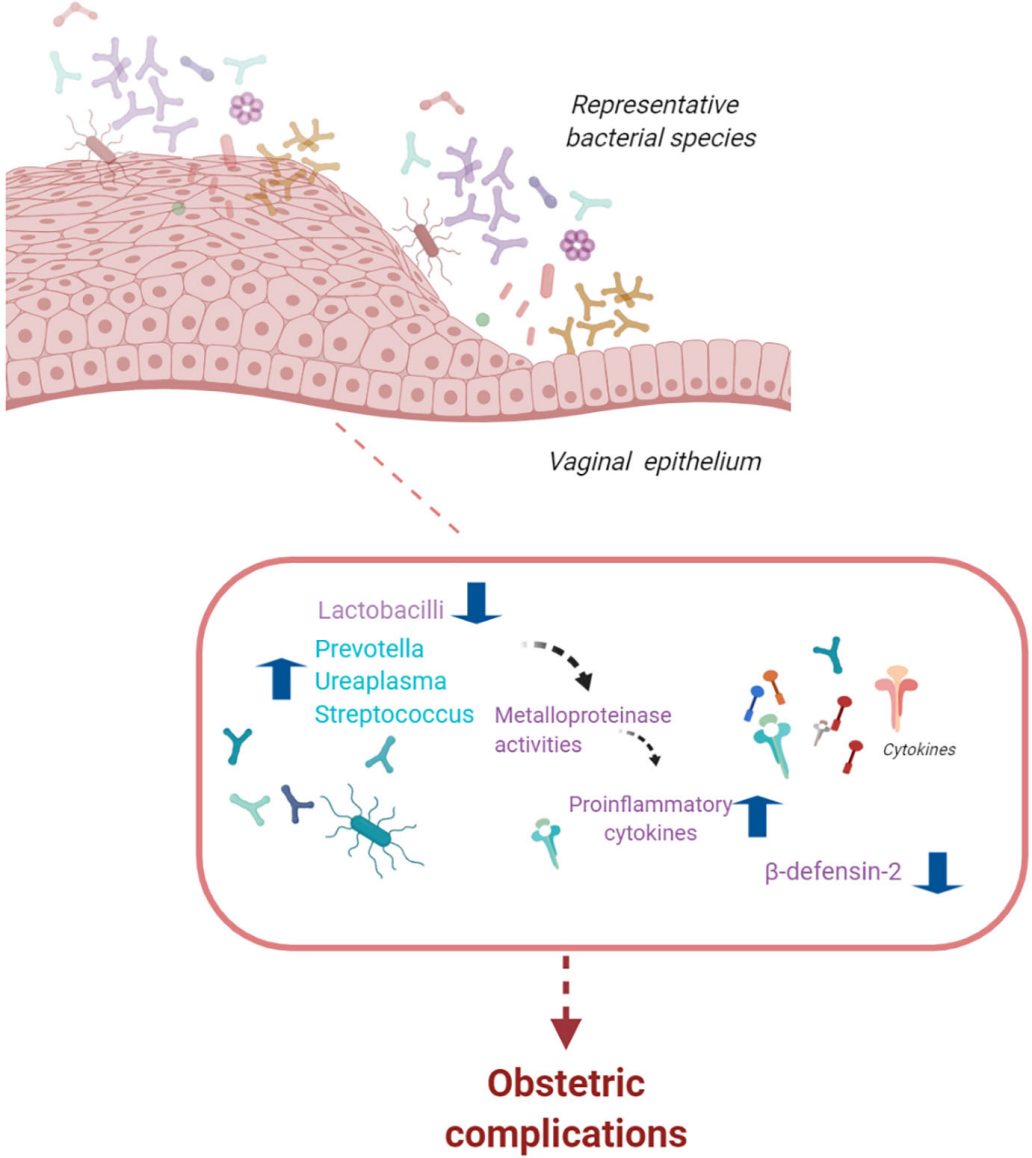
The vaginal microbiota. The vaginal microbiota is composed of a variety of bacterial species. Alterations in lactobacilli dominance and a microbiota with high bacterial diversity are associated with an increased risk of infections, spontaneous preterm birth, and pelvic inflammatory disease (figure created with BioRender.com).

The vaginal microbiota changes throughout a woman’s reproductive life from puberty to menopause with variations during the menstrual cycle (44). In the healthy female reproductive tract, lactobacilli are dominant. One of the key functions of lactobacilli is to activate glycogen metabolism. Glycogen produced by vaginal epithelial cells is transformed into lactic acid, inducing a low vaginal pH (3.8–4.4). This creates an unfavorable environment for the growth of pathogenic bacteria (45). Vaginal dysbiosis, which is linked to inflammatory states, is associated with adverse obstetric outcomes (**Figure 2**) (46). In the presence of dysbiosis, the vaginal microbiota increases the levels of vaginal inflammatory cytokines, which, in turn, increases the risk of spontaneous preterm birth (sPTB) (46–48). However, the debate on the relationship between vaginal dysbiosis and an increased risk of obstetric complications is ongoing. Several studies on the vaginal microbiota and sPTB rely on small sample sizes, primarily because data on vaginal swabs throughout pregnancy are often absent; where they exist, they show limited information on sPTB.

Alterations in lactobacilli dominance are likely to influence a patient’s reproductive potential. In the presence of a microbiota with high bacterial diversity, as in bacterial vaginosis, an increased risk of infections, sPTB, and pelvic inflammatory disease have been observed (47–56). Consistent with these data, an increased risk of sPTB was detected in patients with low levels of *Lactobacillus* and increased bacterial diversity with *Gardnerella vaginalis* and *Mycoplasma* (47–56). Presence of *Lactobacillus* and low bacterial diversity were detected in women with term deliveries (56, 57). *Lactobacillus iners* is a risk factor for sPTB in high-risk patients; however, some studies suggest that the presence of *Lactobacillus crispatus* in the vaginal microbiota is protective against sPTB (56, 57). Because sPTB might be related to pathogenic microbes able to ascend from the vagina, these observations suggest that early characterization of the vaginal microbiota might be a predictive marker for obstetric complications, such as sPTB.

The relationship between the vaginal microbiota and obstetric complications is population-dependent. Women of European ancestry are more likely to harbor a *Lactobacillus*-dominated microbiome, whereas African American women are more likely to exhibit a diverse microbiotic profile. These women are also twice as likely to be diagnosed with bacterial vaginosis and twice as likely to experience preterm birth (53). By comparing African American women with women of European ancestry, Fettweis et al. (53) find that vaginal microbiotic diversity is significantly greater in African American women. In these women, the most common profile was *L*. *iners* followed by *G. vaginalis*, *Candidatus* Lachnocurva vaginae (also known as bacterial vaginosis-associated bacterium 1), and *L*. *crispatus*. In contrast, the most common profile in women of European ancestry was *L*. *crispatus*, followed by *L*. *iners* and *G*. *vaginalis*. These results suggest that there are significant differences in vaginal microbiota related to ancestry (53); such differences might explain the observed prevalence of bacterial vaginosis and preterm birth. Vaginal dysbiosis is associated with increased levels of proinflammatory cytokines (58). Recently, Fettweis et al. (53) observed that levels of the vaginal inflammatory cytokine CXCL10 were related to the *L*. *crispatus*/*L*. *iners* ratio in patients at increased risk of sPTB, indicating a cytokine/*Lactobacillus* ratio as a possible predictive marker for sPTB. However, the difference between preterm and term deliveries cannot be explained only by a lack of *Lactobacillus* species given that many women deliver at term despite lacking *Lactobacillus* species. Conversely, the presence of *Lactobacillus* species does not guarantee a term birth, suggesting that there may be a risk associated with other causes of sPTB. Recently, Elovitz et al. (55) show that immune factors, such as beta-defensin 2, can modulate the risk independently of the presence or absence of *Lactobacillus* species. Indeed, high vaginal levels of beta-defensin 2 have been shown to lower the risk of sPTB. However, the reasons why some women have high or low beta-defensin 2 levels are unknown.

Despite research efforts, sPTB is one of the most common causes of neonatal death and infant mortality with consequences persisting from early childhood into adulthood; this presents families and society with important emotional and financial costs. Existing empirical evidence suggests that future population-specific studies may be able to shed light on the role of the vaginal microbiota, thereby supporting the development of therapeutic strategies. These include immune modulators and microbiome-based therapeutic approaches.

### The Endometrial Microbiota

**Figure 3:**
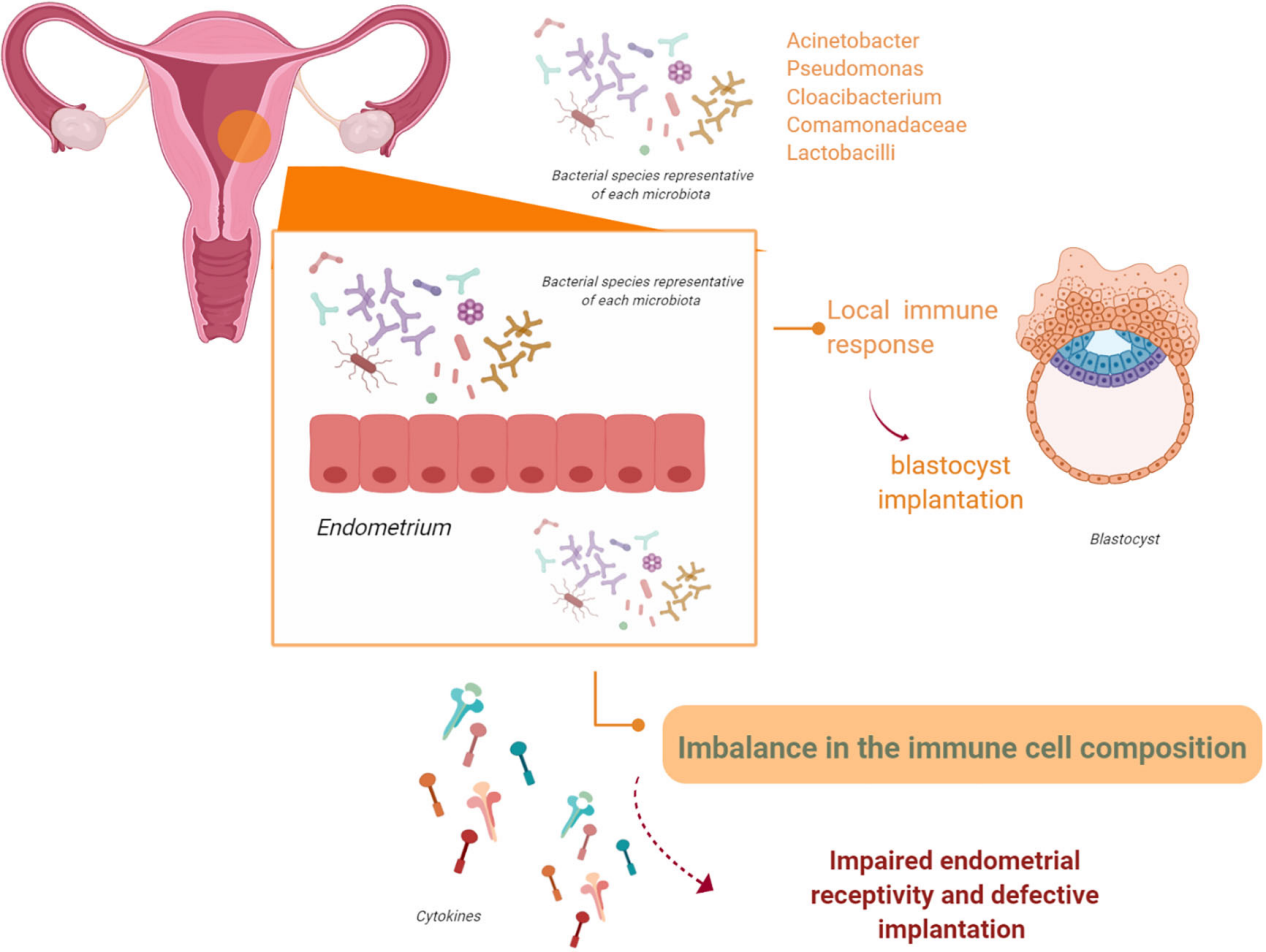
The endometrial microbiota. The endometrium is not a sterile tissue. Resident populations of microorganisms at the endometrial level have been observed. It is possible that these microorganisms might interact with the endometrial epithelium and/or alter endometrial expression of leukocytes and cytokines. Therefore, these events, either in isolation or acting together, may impair endometrial receptivity and affect adequate implantation (figure created with BioRender.com).

The endometrium is a site of immune surveillance where different components from the immune system work together to prevent infections and allow implantation of the blastocyst during pregnancy (59–61). When pregnancy begins, the endometrium undergoes decidualization; modifications in immune cell composition also occur (59–64). The local immune response, influenced by ovarian steroids, is essential for successful blastocyst implantation. Alteration of the endometrial immunological response during pregnancy has been linked to pregnancy complications, such as early pregnancy loss, preterm delivery, PE, and fetal growth restriction (**Figure 3**) (65–69).

The endometrium is not a sterile site. Recently, due to the sequencing of specific regions of bacterial ribosomal RNA, a resident endometrial microbiota and microbiome have been defined (70–73). Early studies on the endometrial microbiota reported the dominance of *Lactobacillus* species. Moreno et al. (74, 75) report an association between low levels of *Lactobacillus* species (<90% *Lactobacillus* with >10% other bacteria) in the endometrial microbiota and poor pregnancy outcomes regarding implantation success and ongoing and term pregnancy rates. A further study describes an endometrial microbiota mainly dominated by *Bacteroides* (76). Nevertheless, the heterogeneous characteristics of the women included in these studies (obstetric history, number of previous term deliveries, demographics, and medical history) limited this research. More recently, the endometrial microbiota obtained from the tip of the transfer catheter in 70 women undergoing *in vitro* fertilization was analyzed (77). In line with other studies, vaginal bacterial *Lactobacillus* species were dominant (>70% abundance). Furthermore, *Corynebacterium*, *Bifidobacterium*, *Staphylococcus*, and *Streptococcus* were observed (73, 78). However, a key limitation of the study was the heterogeneity of the population analyzed.

Winters et al. (79) recently questioned these observations. They suggest that the transcervical catheter collection of endometrial microbiota used in previous research was more prone to contamination. To overcome this, they obtained endometrial samples from hysterectomies and found an endometrial microbiota mainly composed of *Acinetobacter*, *Pseudomonas*, *Cloacibacterium*, and Comamonadaceae. Notably, they report that *Lactobacillus* was rare in the endometrial samples they analyzed. Finally, endometrial bacterial composition was different from that of the vagina. Instead, it was correlated to that of the cervix regarding composition and bacterial load. To date, the role of the endometrial microbiota in female reproduction is not fully understood. Liu et al. (80) try to link endometrial microbiota to chronic endometritis (CE) (81–84). The gold standard for the diagnosis of CE relies on histological identification of plasma cells in the endometrial stroma (84). The impact of CE on reproductive capacity is not well known. The prevalence of CE in the general population ranges from 0.8% to 19%. This percentage reaches 30%–45% in patients who are infertile or experience recurrent pregnancy loss (85–90). The mechanisms involved in CE-related poor pregnancy outcomes include imbalance in immune cell composition in the endometrium, lower response to steroid hormones, impairment in glycodelin secretion, and altered expression of pinopodes (91–94).

Liu et al. (80) compare the endometrial microbiota of infertile women with and without CE. They obtained endometrial biopsies and postovulatory-phase endometrial fluid from 130 infertile women. They found that CE was strictly associated with an increased proportion of non-*Lactobacillus* bacterial taxa in the endometrial cavity although the mechanisms underlying such a correlation are unknown.

Additionally, several recent studies suggest the presence of a resident microbiota in the endometrium (95–97). Yet studies that evaluate the role of the endometrial microbiota on reproductive health are in their infancy. We speculate that the endometrial microbiota may interact with the endometrial epithelium and endometrial immune cells, ultimately resulting in impaired endometrial receptivity and defective implantation.

To date, much remains to be understood regarding the ability of bacteria to colonize the endometrium and/or establish a commensal/pathogenic relationship with intrauterine tissues (98, 99).

## Conclusions

The human microbiota plays a central role in health and female morbidity. Therefore, classifying women based on bacterial patterns would allow a personalized, microbiota-based diagnosis, which could then be used to develop personalized therapies for disease prevention and personalized treatments. These treatments could be used to modulate the composition of the microbiota. Women planning to have a family could be asked to consume specific nutrients, foods, and probiotics as well as making appropriate lifestyle changes. Pharmaceutical intervention is another useful adjunct.

## Author Contributions

NS and SD’I conceived and designed the study. NS, AO, and SD’I drafted and revised the article where appropriate. MS prepared the figures. NS, AO, CT, PV, AG, GS, and SD’I carried out the final revision of the manuscript. All authors contributed to the article and approved the submitted version.

## Funding

This research was supported by funding from Università Cattolica del Sacro Cuore (D12019,2018-2019). The funding institution had no influence on study design, manuscript preparation, or the decision to publish.

## Conflict of Interest

The authors declare that the research was conducted in the absence of any commercial or financial relationships that could be construed as a potential conflict of interest.
